# Supplementary material for: Meanders as a scaling motif for understanding of floodplain soil microbiome and biogeochemical potential at the watershed scale
Source: Microbiome. 2021 May 22;9:121. doi: 10.1186/s40168-020-00957-z (PMC8141241; doi:10.1186/s40168-020-00957-z)
Supplement: Supplementary file 4 — Additional file 3: Figure S1. Rank abundance curve of rpL6 scaffolds (centroids for sequences clustered at 97.5 % nucleotide identity) and their taxonomic assignment. Black ticks represent clusters with scaffolds that were binned in genomes. Clusters with < 5 sequences were combined in “others”. The taxonomic affiliation presented here corresponds to that of the Genome Taxonomy Database (GTDB release 05-RS95), while the taxonomy of the genomes was assigned based on phylogenetic placement in a tree of 14 concatenated ribosomal proteins. There may be discrepancies in the taxonomic assignments between the single rpL6 genes and the genomes, arising from the use of a different method to generate this figure. For example, Gammaproteobacteria in GTDB (https://gtdb.ecogenomic.org/) include Betaproteobacteria, and Deltaproteobacteria in NCBI include Myxococcota and Desulfobacterota, which are separate clades in GTDB. Scaffolds from multiple CPR (Patescibacteria) were not classified by GTDB and have been grouped as “Unk” or unclassified in this figure. Figure S2. Percent of samples within each floodplain where a genome was detected at the sub-species level (98% ANI). Presence or absence was determined based on Hellinger transformed abundance (average coverage ≥ 0.01). (a) Detection regardless of where the genome was reconstructed from. (b) Detection according to floodplain of origin. Genomes were detected in a higher number of samples from a given floodplain if they were reconstructed from a sample within that floodplain. Figure S3. Concatenated ribosomal proteins IQ-TREE of Betaproteobacteria at the sub-species level (98% ANI) and ~ 1540 reference genomes from the NCBI. East River Betaproteobacteria are shown in bold magenta font (from this study) and violet font (from [49]). Some environmental sequences related to East River Betaproteobacteria are highlighted in orange, and next to the accession number is the environment of origin. Clades at the family level follow GTDB tax [file 40168_2020_957_MOESM4_ESM.zip › Figure_S3_Betaproteobacteria_98ANI_IQtree.pdf]

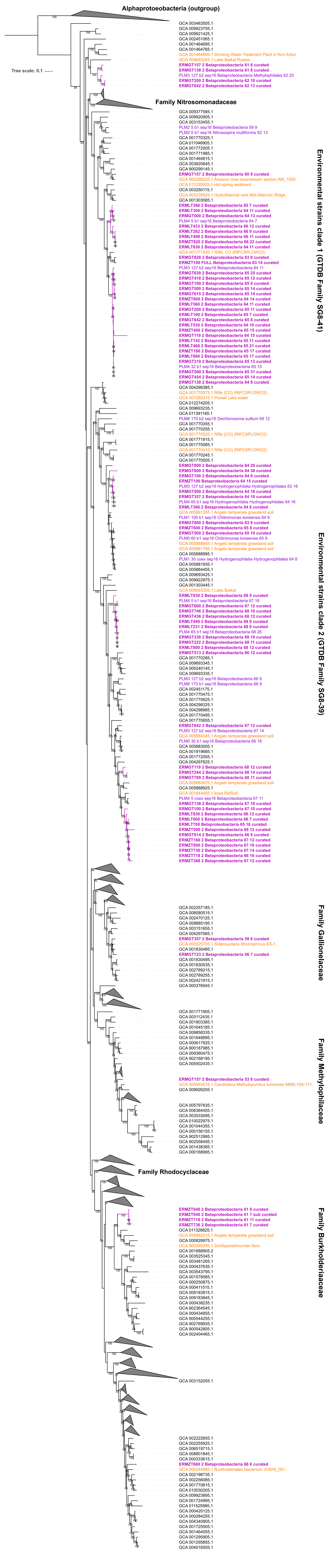
Alphaprotoeobacteria (outgroup)
Tree scale: 0.1
Family Nitrosomonadaceae
Environmental strains clade 1 (GTDB Family SG8-41)
Environmental strains clade 2 (GTDB Family SG8-39)
Family Gallionellaceae
Family Methylophilaceae
Family Rhodocyclaceae
Family Burkholderiaceae
