# Supplementary material for: Meanders as a scaling motif for understanding of floodplain soil microbiome and biogeochemical potential at the watershed scale
Source: Microbiome. 2021 May 22;9:121. doi: 10.1186/s40168-020-00957-z (PMC8141241; doi:10.1186/s40168-020-00957-z)
Supplement: Supplementary file 4 — Additional file 3: Figure S1. Rank abundance curve of rpL6 scaffolds (centroids for sequences clustered at 97.5 % nucleotide identity) and their taxonomic assignment. Black ticks represent clusters with scaffolds that were binned in genomes. Clusters with < 5 sequences were combined in “others”. The taxonomic affiliation presented here corresponds to that of the Genome Taxonomy Database (GTDB release 05-RS95), while the taxonomy of the genomes was assigned based on phylogenetic placement in a tree of 14 concatenated ribosomal proteins. There may be discrepancies in the taxonomic assignments between the single rpL6 genes and the genomes, arising from the use of a different method to generate this figure. For example, Gammaproteobacteria in GTDB (https://gtdb.ecogenomic.org/) include Betaproteobacteria, and Deltaproteobacteria in NCBI include Myxococcota and Desulfobacterota, which are separate clades in GTDB. Scaffolds from multiple CPR (Patescibacteria) were not classified by GTDB and have been grouped as “Unk” or unclassified in this figure. Figure S2. Percent of samples within each floodplain where a genome was detected at the sub-species level (98% ANI). Presence or absence was determined based on Hellinger transformed abundance (average coverage ≥ 0.01). (a) Detection regardless of where the genome was reconstructed from. (b) Detection according to floodplain of origin. Genomes were detected in a higher number of samples from a given floodplain if they were reconstructed from a sample within that floodplain. Figure S3. Concatenated ribosomal proteins IQ-TREE of Betaproteobacteria at the sub-species level (98% ANI) and ~ 1540 reference genomes from the NCBI. East River Betaproteobacteria are shown in bold magenta font (from this study) and violet font (from [49]). Some environmental sequences related to East River Betaproteobacteria are highlighted in orange, and next to the accession number is the environment of origin. Clades at the family level follow GTDB tax [file 40168_2020_957_MOESM4_ESM.zip › Supplementary_Figures.pdf]

**Supplementary Information for:**

# Meanders as a scaling motif for understanding of floodplain soil microbiome and biogeochemical potential at the watershed scale

Paula B. Matheus Carnevali[1], Adi Lavy[1], Alex D. Thomas[2], Alexander Crits-Christoph[3], Spencer Diamond[1], Raphaël Méheust[1,4], Matthew R. Olm[3,5], Allison Sharrar[1], Shufei Lei[1], Wenmin Dong[6], Nicola Falco[6], Nicholas Bouskill[6], Michelle E. Newcomer[6], Peter Nico[6], Haruko Wainwright[6], Dipankar Dwivedi[6], Kenneth H. Williams[6], Susan Hubbard[6], Jillian F. Banfield[1,2,3,4,5,6,7*].

[1]Department of Earth and Planetary Science, University of California, Berkeley, CA, USA.
[2]Department of Environmental Science, Policy, and Management, University of California, Berkeley, CA, USA.
[3]Department of Plant and Microbial Biology, University of California, Berkeley, CA, USA.
[4]Innovative Genomics Institute, Berkley, CA, USA.
[5]Current affiliation: Department of Microbiology and Immunology, Stanford University, Palo Alto, CA, USA.
[6]Earth and Environmental Sciences Area, Lawrence Berkeley National Laboratory, Berkeley, CA, USA
[7]Chan Zuckerberg Biohub, San Francisco, CA, USA.

*Corresponding author: jbanfield@berkeley.edu

File contents:
Supplementary figures and legends S1, S2, S4-S9
Supplementary legend for figure S3

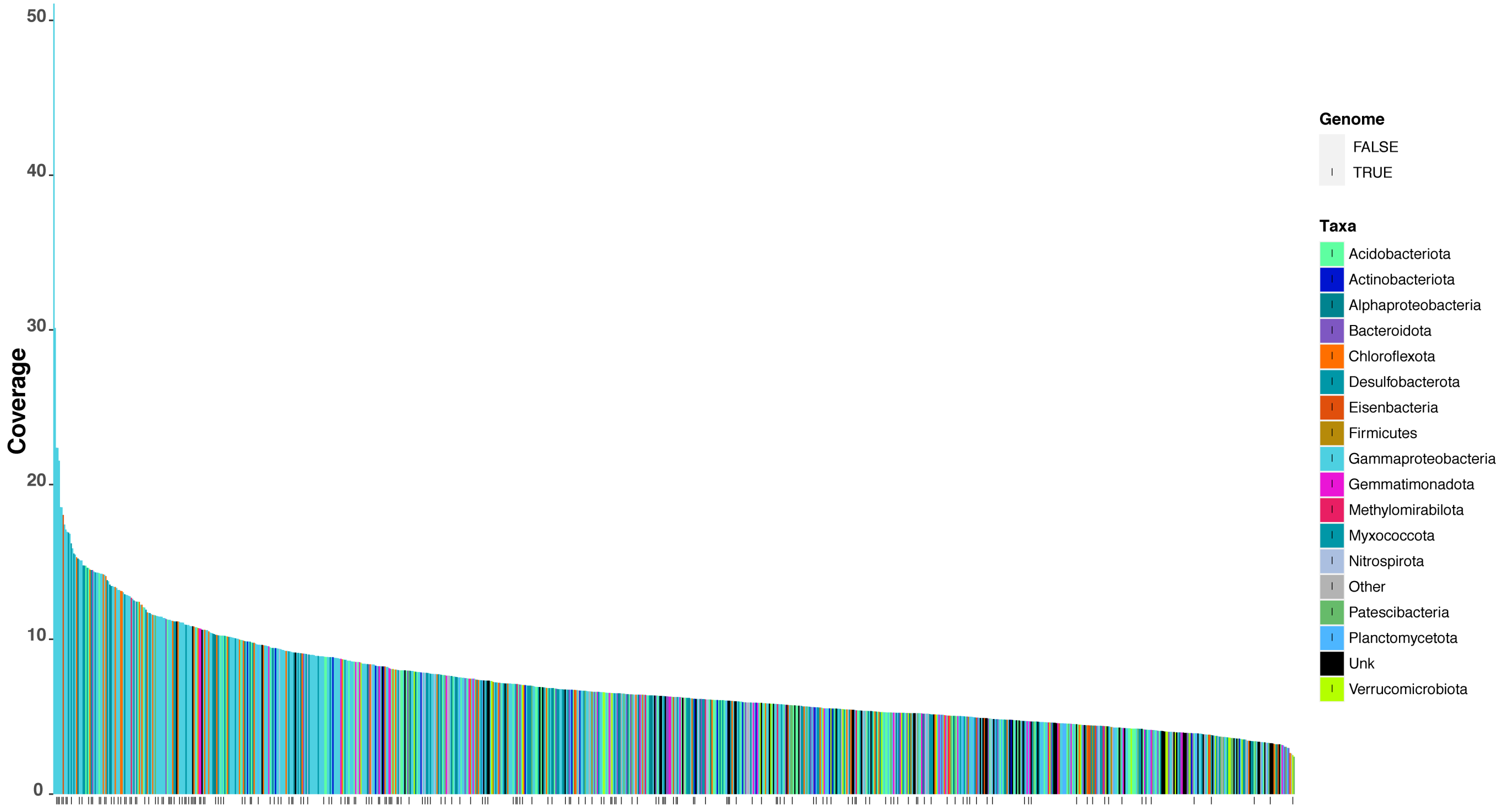


**Figure S1.** Rank abundance curve of rpL6 scaffolds (centroids for sequences clustered at 97.5 % nucleotide identity) and their taxonomic assignment. Black ticks represent clusters with scaffolds that were binned in genomes. Clusters with < 5 sequences were combined in "others". The taxonomic affiliation presented here corresponds to that of the Genome Taxonomy Database (GTDB release 05-RS95), while the taxonomy of the genomes was assigned based on phylogenetic placement in a tree of 14 concatenated ribosomal proteins. There may be discrepancies in the taxonomic assignments between the single rpL6 genes and the genomes, arising from the use of a different method to generate this figure. For example, Gammaproteobacteria in GTDB (https://gtdb.ecogenomic.org/) include Betaproteobacteria, and Deltaproteobacteria in NCBI include Myxococcota and Desulfobacterota, which are separate clades in GTDB. Scaffolds from multiple CPR (Patescibacteria) were not classified by GTDB and have been grouped as "Unk" or unclassified in this figure.

a

b

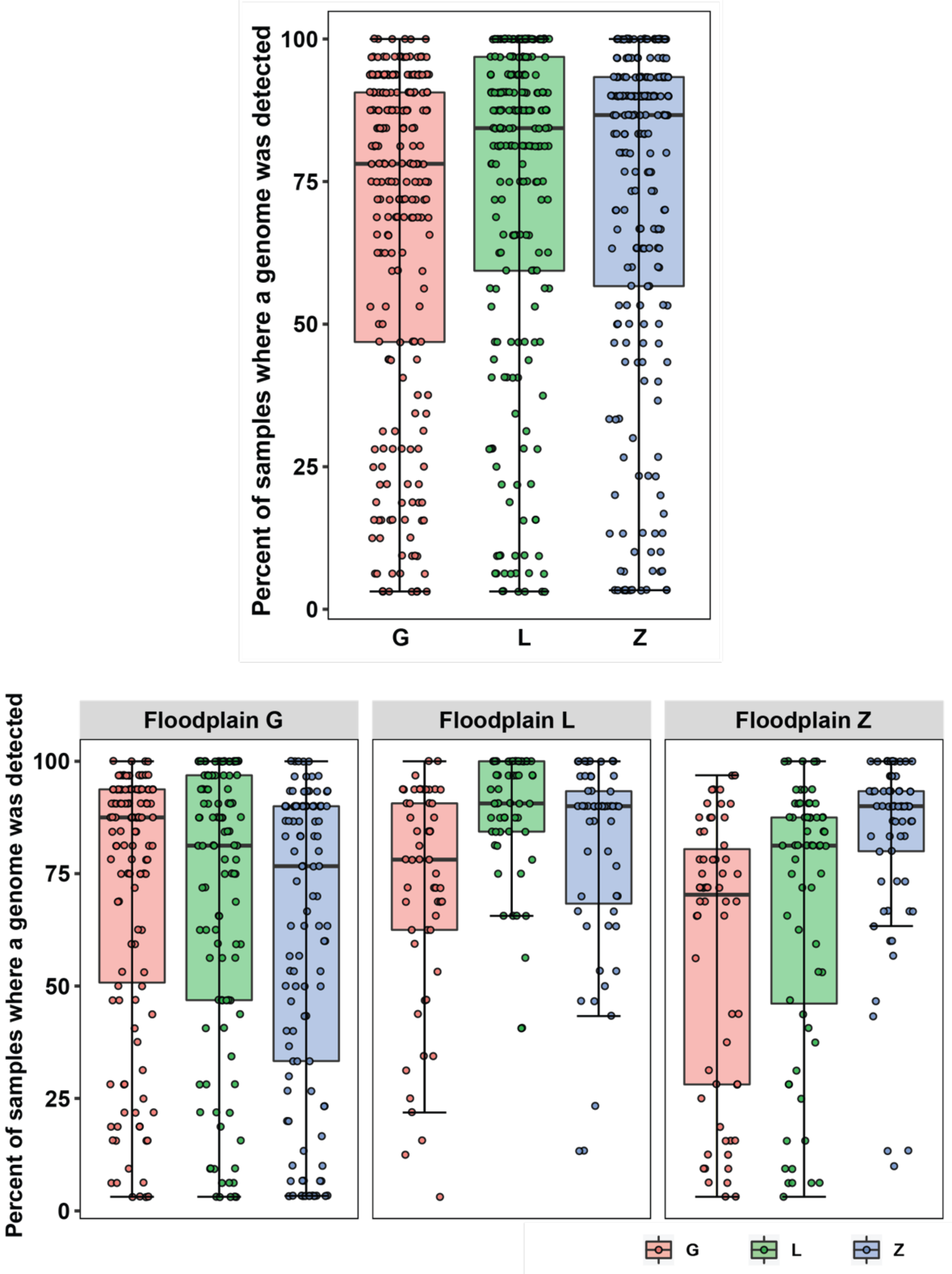


**Figure S2.** Percent of samples within each floodplain where a genome was detected at the sub-species level (98% ANI). Presence or absence was determined based on Hellinger transformed abundance (average coverage ≥ 0.01). (**a**) Detection regardless of where the genome was reconstructed from. (**b**) Detection according to floodplain of origin. Genomes were detected in a higher number of samples from a given floodplain if they were reconstructed from a sample within that floodplain.

**See separate pdf file (Additional file 3) and Data S1 (Additional file 5)**

**Figure S3.** Concatenated ribosomal proteins IQ-TREE of Betaproteobacteria at the sub-species level (98% ANI) and ~ 1540 reference genomes from the NCBI. East River Betaproteobacteria are shown in bold magenta font (from this study) and violet font (from [1]). Some environmental sequences related to East River Betaproteobacteria are highlighted in orange, and next to the accession number is the environment of origin. Clades at the family level follow GTDB taxonomy [2] for additional reference.

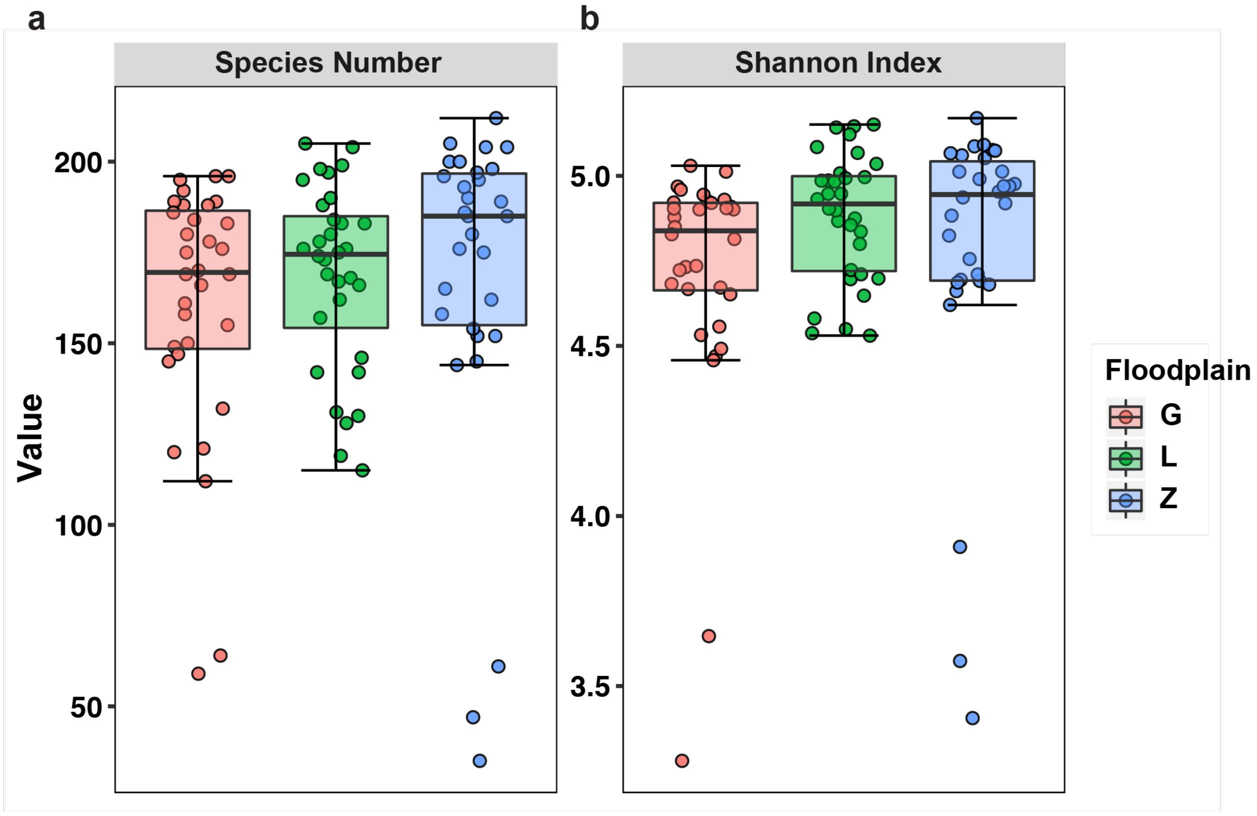


**Figure S4.** Diversity indices calculated for a set of representative genomes at the sub-species level (98% ANI). (**a**) Species Number. (**b**) Shannon Index.

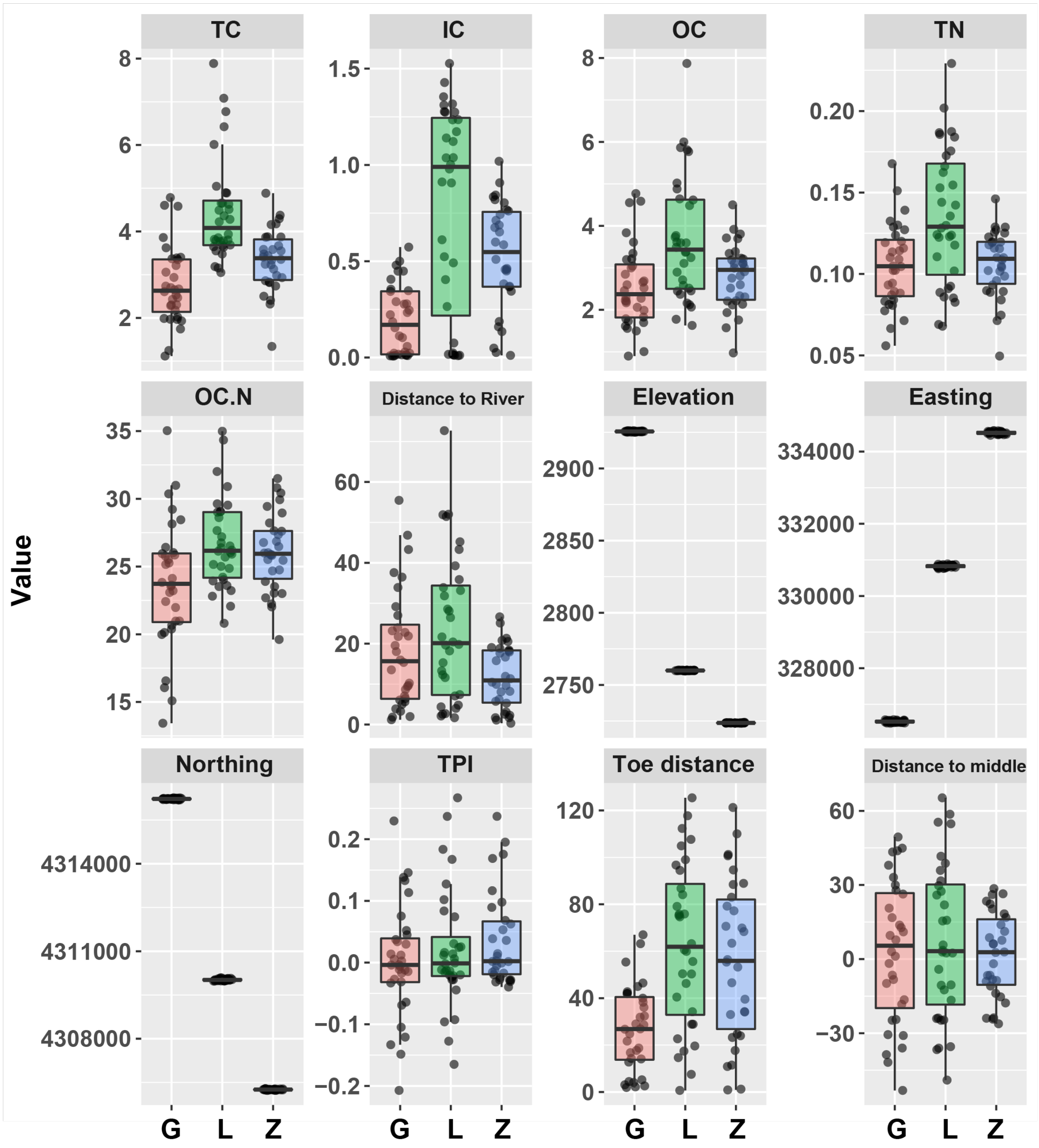


**Figure S5.** Environmental variables used in fourth corner analysis: solid face chemistry from soil samples collected in 2015 including total carbon (TC; %), inorganic carbon (IC; %), organic carbon (OC; %), total nitrogen (TN; %), total carbon to total nitrogen ratio (OC:N) and measures associated with sample site locations: distance to river, elevation, easting, northing, topographic position index (TPI), and distance to the inner bank edge (or toe distance) and distance to middle of the meander-bound floodplain.

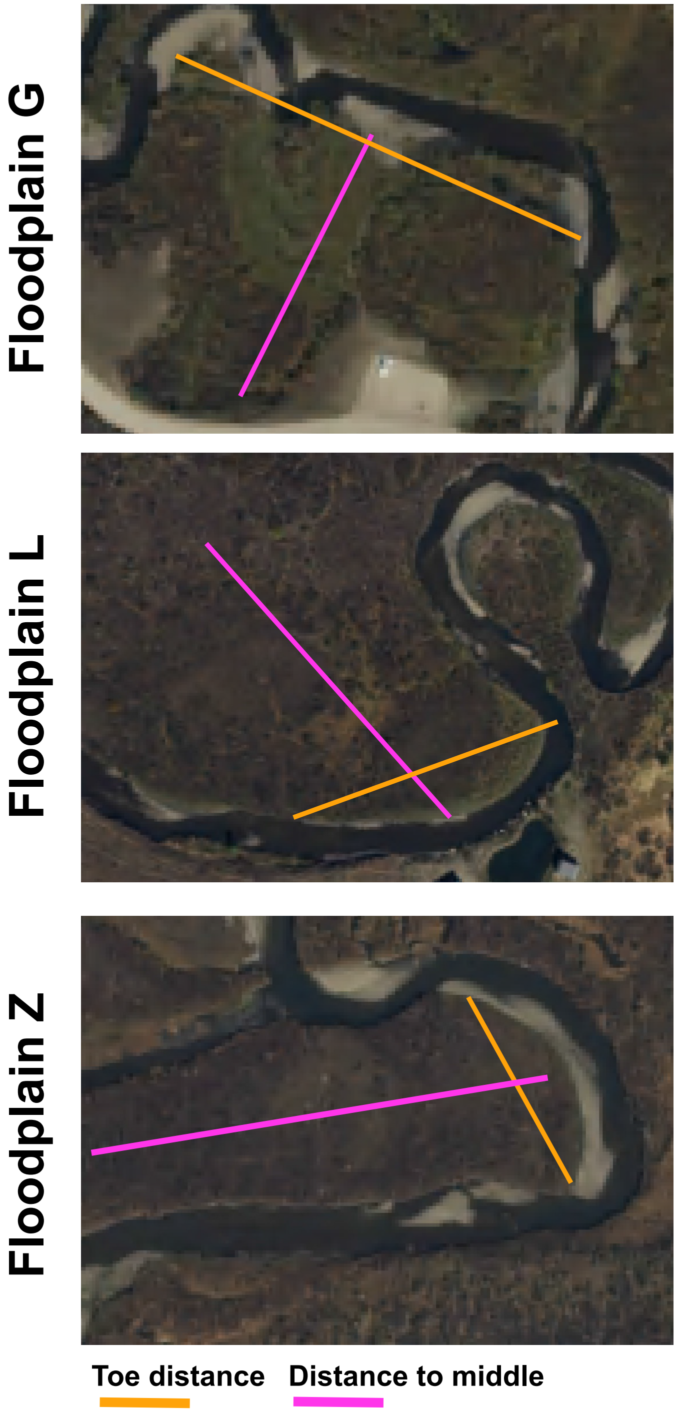


**Figure S6.** Diagram representing imaginary lines used to determine distance to the middle of the meander-bound floodplain and distance to the inner bank edge (toe distance) as alternative measures of samples position on the floodplains.

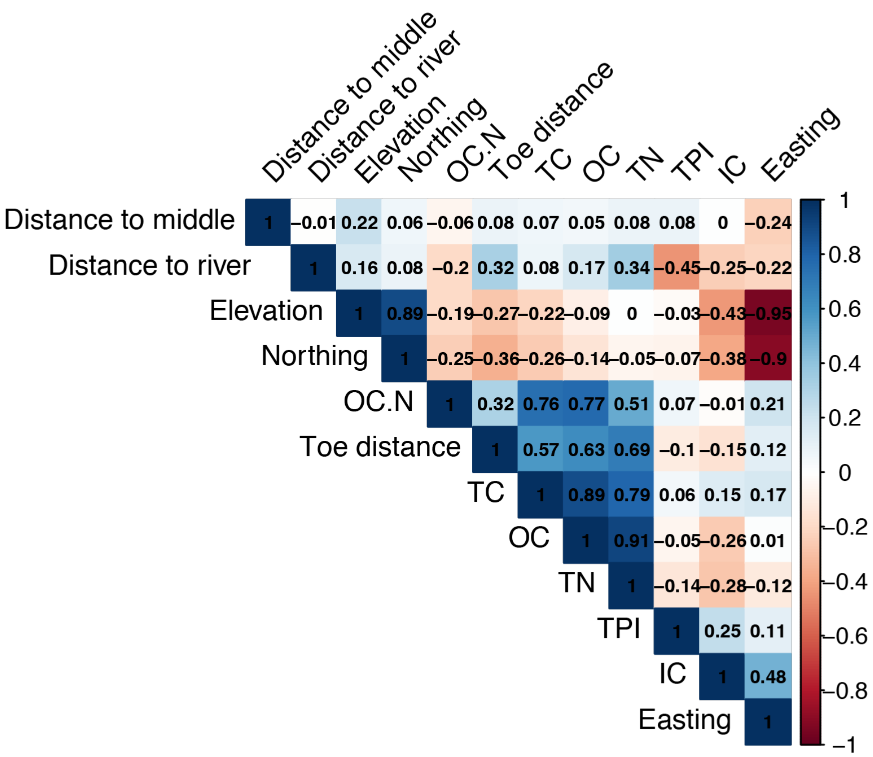


**Figure S7.** Spearman's correlation among environmental variables (2015).

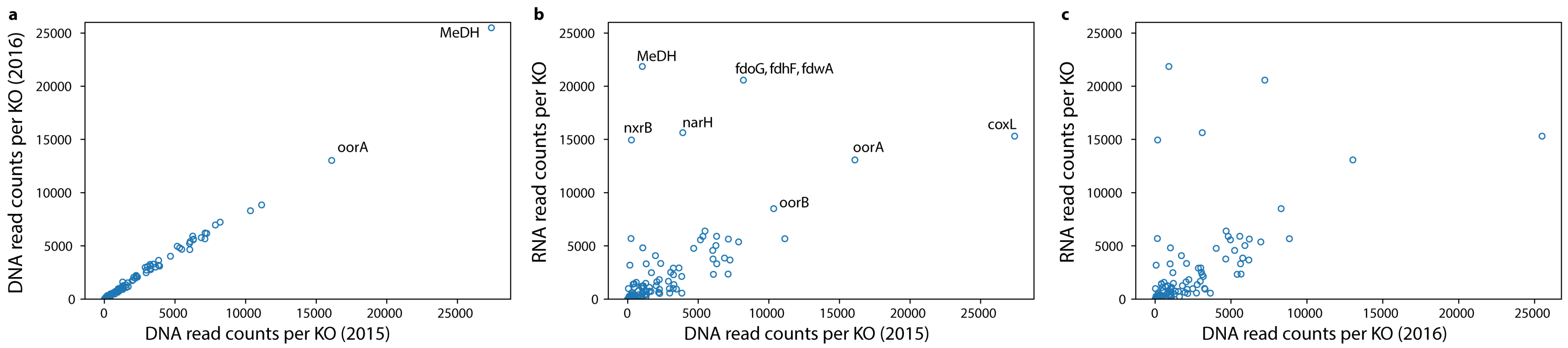


**Figure S8.** Number of reads mapped to genes encoding geochemically relevant functions in the species-level set of 215 genomes. (**a**) Number of DNA reads mapped from 2015 metagenomes and from 2016 metagenomes. This plot shows a high degree of correspondence between the two different years. (**b**) Number of RNA and DNA reads mapped from 2016 metatranscriptomes and 2015 metagenomes obtained from the same sampling sites. (**c**) Number of RNA and DNA reads from 2016 metatranscriptomes and metagenomes from the same subset of sampling sites. These plots show that in some cases there are high numbers of RNA reads mapping to low abundance genes (represented by low number of mapped DNA reads). Most obvious examples on figures b and c: MeDH: methanol dehydrogenase. *nxrB*: nitrite oxidoreductase subunit B. *narH*: membrane-bound nitrate reductase ($H^+$ - translocating). *fdoG, fdhF, fdwA*: $NAD^+$-dependent formate dehydrogenase major subunit. *oorAB*: 2-oxoglutarate/2-oxoacid ferredoxin oxidoreductase subunits alpha and beta. *coxL*: aerobic carbon monoxide dehydrogenase large subunit.

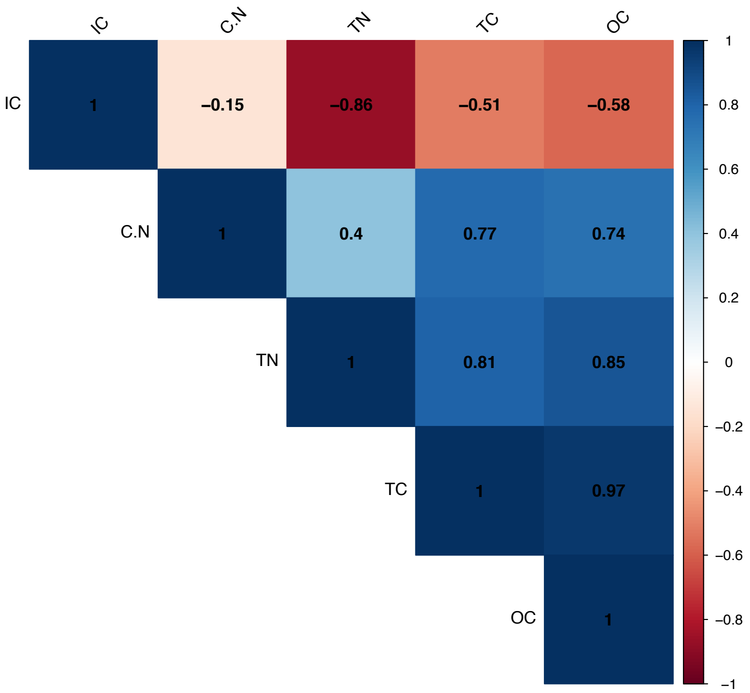


**Figure S9.** Spearman's correlation among environmental variables (2016).

**References**


1. Lavy A*, et al.* Taxonomically and metabolically distinct microbial communities with depth and across a hillslope to riparian zone transect. *bioRxiv*, 768572 (2019).

2. Parks DH, Chuvochina M, Chaumeil PA, Rinke C, Mussig AJ, Hugenholtz P. A complete domain-to-species taxonomy for Bacteria and Archaea. *Nat Biotechnol*, (2020).
